# Schizophrenia detection via lobe-wise and overall EEG features using VMD and bayesian-optimized machine learning models

**DOI:** 10.3389/fnins.2026.1753779

**Published:** 2026-03-05

**Authors:** Gandham Sai Sravanthi, Lakhan Dev Sharma

**Affiliations:** School of Electronics Engineering, VIT-AP University, Amaravati, Andhra Pradesh, India

**Keywords:** EEG, feature extraction, optimized ML, schizophrenia, VMD

## Abstract

Schizophrenia (SCH) is a chronic and severe mental disorder that leads to significant cognitive and neurophysiological impairments, affecting daily life. Early diagnosis remains challenging as it relies on the manifestation of symptoms that develop over time. Electroencephalography (EEG), which measures brain activity, provides a promising avenue for early detection. In this study, two EEG datasets—the Mental Health Research Center (MHRC) and the Repository for Open Data (RepOD)—were employed to detect SCH. EEG signals were segmented into 8-second durations and decomposed using Variational Mode Decomposition (VMD) into 10 Intrinsic Mode Functions (IMFs). Multi-domain features extracted from IMFs were classified using nine machine learning (ML) and seven optimized ML (OML) classifiers. The proposed method achieved an accuracy (Ac) of 96.7% for the MHRC dataset using the Optimizable KNN classifier and 99.0% for the RepOD dataset using the Optimizable Ensemble classifier. To prevent data leakage, a strict subject-wise Leave-One-Out Cross-Validation (LOOCV) strategy was employed. Lobe-wise analysis showed that the frontal lobe achieved accuracies of 91.2% for MHRC using the Optimizable Ensemble and 99.4% for RepOD using the Optimizable Neural Network, with the temporal lobe also showing strong discriminative power. These findings align with established evidence of frontal–temporal dysconnectivity in SCH. Overall, the proposed VMD + OML framework offers a computationally efficient and clinically interpretable solution for early SCH detection using EEG signals.

## 1 Introduction

The electroencephalogram (EEG), often termed brain waves, represents one of the most powerful and noninvasive tools for assessing neural activity in real time. EEG records the brain's electrical fluctuations using electrodes placed across the scalp, capturing the oscillatory firing patterns of cortical neurons. These oscillations are distributed across distinct frequency bands such as Delta (0.5–4 Hz), Theta (4–8 Hz), Alpha (8–13 Hz), and Beta (>13 Hz), each associated with specific cognitive and physiological functions (Rangayyan, 2015; Babiloni et al., 2020). For instance, alpha rhythms dominate during relaxed wakefulness with eyes closed, theta activity arises in early sleep and meditative states, and delta waves occur during deep sleep. Conversely, beta activity is linked to alertness and mental engagement. Deviations from these normal rhythmic patterns may signify neural dysfunction or neuropathological conditions (Miller, 2007; Dang-Vu et al., 2008; Lanillos et al., 2020).

EEG abnormalities are key indicators for diagnosing neurological and psychiatric disorders, including epilepsy, depression, Alzheimer's disease, and Schizophrenia (SCH) (Cascella et al., 2009; Yasin et al., 2021). SCH is a chronic psychiatric disorder affecting 1% of the population, typically emerging in late adolescence or early adulthood, and is characterized by hallucinations, delusions, cognitive impairments, and social withdrawal (Aggernaes, 1994; Tandon et al., 2008; McGrath et al., 2008). Despite extensive research, its neurobiological mechanisms remain unclear, and diagnosis still relies largely on behavioral observation and patient self-reporting (McGlashan, 1999; Nieuwenhuis et al., 2012). EEG studies in SCH reveal disrupted functional connectivity, abnormal synchronization, reduced complexity, and irregular power spectral density (PSD) in delta, theta, and gamma bands (Winterer et al., 2021; Kim et al., 2022), highlighting its potential as an objective biomarker. However, the nonlinear and non-stationary nature of EEG signals limits conventional spectral methods such as FFT or STFT.

Adaptive decomposition and feature extraction provide solutions for capturing these abnormalities. Variational Mode Decomposition (VMD) decomposes EEG into narrow-band intrinsic mode functions (IMFs), avoiding mode-mixing problems common in Empirical Mode Decomposition (EMD) (Dragomiretskiy and Zosso, 2014; Praveen et al., 2020; Kumar and Ghosh, 2023), and effectively captures nonlinear patterns and localized frequency shifts. Feature extraction, particularly entropy-based measures—Differential Entropy (DE), Approximate Entropy (ApEn), Sample Entropy (SampEn), Tsallis Entropy (TE), and Higuchi Fractal Dimension (HFD)—quantifies signal irregularity and complexity, which are altered in SCH (Ahmadlou et al., 2012; Dauwels et al., 2020; Hazarika et al., 2019; Richman and Moorman, 2000; Higuchi, 1988; Wang et al., 2022). When combined with statistical parameters such as skewness, kurtosis, and PSD, these features provide a comprehensive representation of EEG dynamics, supporting automated computational diagnosis of SCH.

Moreover, recent studies have demonstrated the effectiveness of adaptive signal decomposition for biomedical signal analysis. Khan and Pachori (2024) and Khan and Pachori (2025) applied multivariate Fourier–Bessel–based empirical wavelet transform (MVFBSE-EWT) to cardiac VCG signals for bundle branch block and posterior myocardial infarction detection, showing that appropriate intrinsic mode selection significantly enhances diagnostic accuracy. Similarly, Khan et al. (2021) employed empirical mode decomposition for lung sound analysis, highlighting that excessive decomposition introduces redundancy. Moorthy et al. (2024) further demonstrated that controlled multilevel decomposition improves feature discriminability in brain tumor identification while avoiding overfitting. Collectively, these works emphasize that selecting a moderate number of intrinsic modes is critical for optimal biomedical signal analysis performance. An EEG classification framework using empirical wavelet transform and amplitude envelope features achieved 98.67% accuracy with KNN using only two features, enabling real-time seizure detection (Khan and Pachori, 2022). Motivated by these findings, this study adopts a controlled VMD-based decomposition strategy to ensure optimal representation of EEG signals for accurate SCH detection.

Machine learning (ML) has emerged as an indispensable tool for decoding such complex EEG feature spaces. Traditional ML algorithms, including Support Vector Machines (SVM), K-Nearest Neighbors (KNN), Decision Trees, and Naive Bayes, have been extensively applied for EEG classification tasks (Tzimourta et al., 2020; Cai et al., 2023). More sophisticated ensemble models, such as Bagged Trees and Boosted Classifiers, can integrate weak learners to achieve superior generalization performance. Despite their success, model performance is highly dependent on hyperparameter tuning. optimization provides a systematic approach for finding optimal model configurations while reducing overfitting and improving classification accuracy (Zhang et al., 2021; Prabhakar et al., 2020). In contrast, deep learning (DL) approaches including Convolutional Neural Networks (CNNs) and recurrent architectures have demonstrated exceptional feature learning capabilities but often require large datasets and longer training durations, making them less practical for limited EEG datasets (Ahmedt-Aristizabal et al., 2020; Li et al., 2022; Jiang et al., 2019).

Several prior studies have explored EEG-based SCH classification using ML and DL techniques. For example, Zhang et al. (2021) employed random forests on event-related potential (ERP) features, achieving 81% accuracy, while Ahmedt-Aristizabal et al. (2020) used SVM and kNN on auditory-evoked EEG data, obtaining 72% accuracy after applying CNN-based enhancement. Buettner et al. (2019) reported a 71% classification rate using random forest classifiers trained on resting-state EEG band power features, whereas Tikka et al. (2020) achieved 79% using 12 statistical features across five frequency bands. More recent studies using nonlinear dynamics and entropy measures, such as those by Jahmunah et al. (2019) and Prabhakar et al. (2020), have achieved accuracies exceeding 92% through SVM-based classifiers, highlighting the promise of complexity-based EEG analysis in SCH detection.

Building upon this foundation, the present study introduces a hybrid EEG-based SCH detection framework that integrates Variational Mode Decomposition (VMD), entropy and fractal features, and optimized ML classifiers. Unlike prior work that relied on a single feature domain or fixed model configurations, this approach captures the multiscale nonlinear dynamics of EEG signals and systematically tunes model parameters to enhance generalization. Two independent and publicly available EEG datasets the Mental Health Research Center (MHRC) and the Repository for Open Data (RepOD) are employed for validation to ensure reproducibility and obustness.

The primary contributions of this study can be summarized as follows:

- A VMD decomposition is applied to EEG signals to extract IMFs associated with SCH-related neural dysregulation.
- Multi-domain features (DE, ApEn, SampEn, TE, PSD, HFD, skewness, and kurtosis) are computed to quantify EEG irregularity and complexity.
- A suite of ML classifiers including SVM, KNN, Ensemble, and Neural Networks is evaluated, followed by optimization to identify the better-performing configurations.
- Lobe-wise classification analysis is performed across the frontal, central, parietal, temporal, and occipital regions, revealing that the frontal and central lobes exhibit the better discriminative capability, with accuracies of 91.2% and 89.4% on the MHRC dataset, and 99.4% and 98.5% on the RepOD dataset, respectively.
- The proposed framework achieves an overall accuracy of 96.7% on the MHRC dataset and 99.0% on the RepOD dataset, outperforming existing EEG-based SCH detection methods.

This framework highlights the diagnostic potential of VMD+OML fusion in identifying SCH with better precision, paving the way for clinically interpretable and data-driven diagnostic systems.

## 2 Datasets

In this research, two publicly available EEG datasets were employed to validate the proposed SCH detection framework: the Mental Health Research Center (MHRC) dataset and the Repository for Open Data (RepOD) dataset, which are described in the following subsections.

### 2.1 MHRC dataset

**Figure 1:**
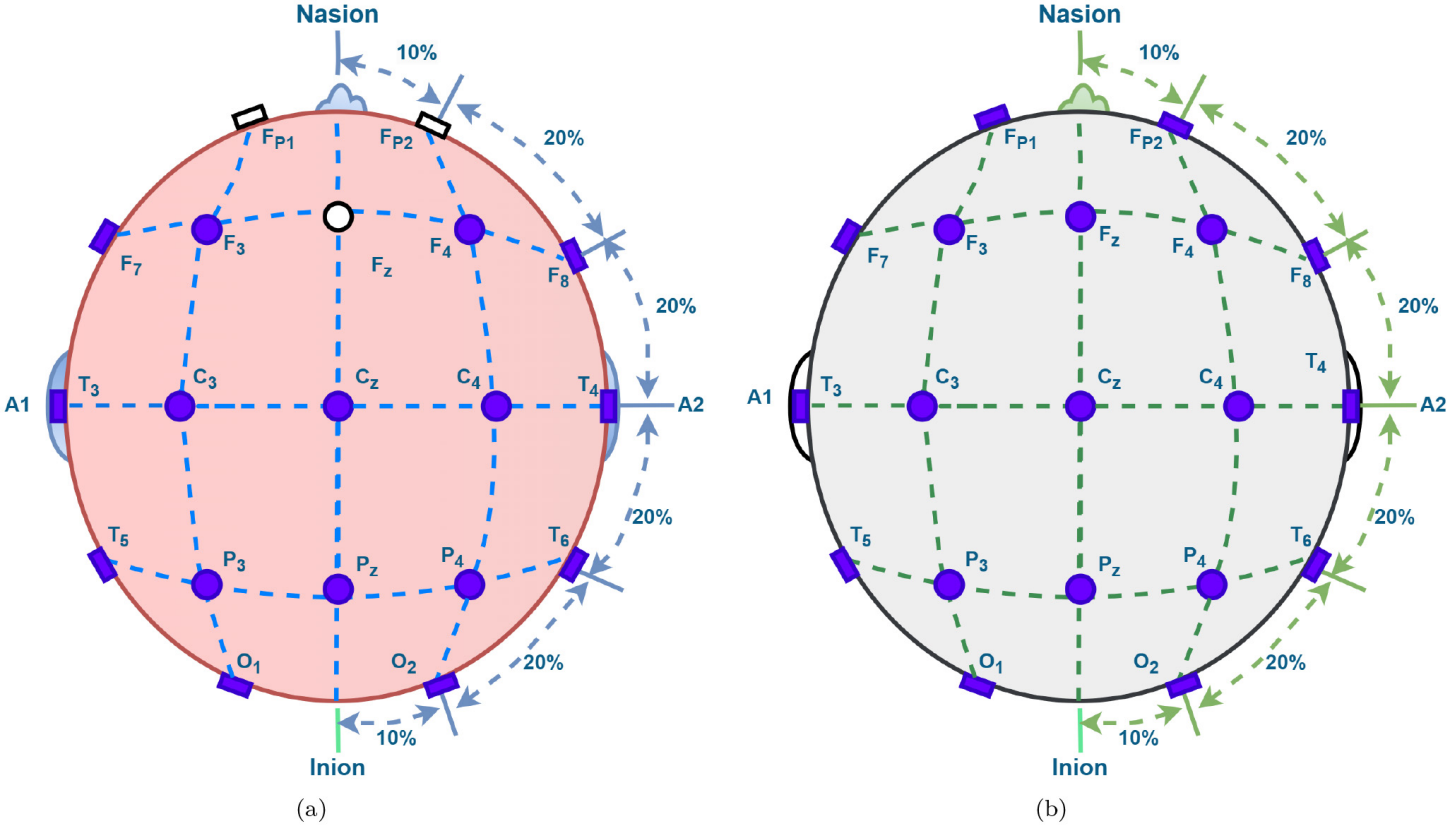
10–20 electrode placement system for EEG recordings (violet color leads are used). **(a)** MHRC. **(b)** RepOD.

The Mental Health Research Center (MHRC) dataset was collected in Moscow and includes EEG recordings from 84 adolescent participants, comprising 39 healthy controls and 45 males diagnosed with childhood SCH or schizoaffective disorder, as confirmed by expert psychiatrists. The participants ages ranged from approximately 10 years 8 months to 14 years, with an overall mean age of about 12 years and 3 months across both groups. EEG signals were recorded using 16 electrodes (O1, O2, P3, P4, Pz, T5, T6, C3, C4, Cz, T3, T4, F3, F4, F7, and F8) arranged according to the standard 10–20 electrode placement system, as shown in Figure 1a. Recordings were performed while subjects were awake, relaxed, and had their eyes closed to minimize artifacts, and each session captured 1-minute segments per electrode at a sampling rate of 128 Hz (MHRC). This well-structured dataset provides a valuable foundation for analyzing neural patterns associated with SCH.

### 2.2 RepOD dataset

The second dataset was obtained from the Repository for Open Data (RepOD) (2017), derived from the seminal work of Olejarczyk and Jernajczyk (2017a). This publicly available dataset contains age and sex-matched EEG recordings from individuals diagnosed with paranoid SCH (ICD-10/F20.0) and healthy control subjects. Each EEG recording was collected during a 15-minute session using the standard 10–20 electrode placement system comprising 19 channels (Fp1, Fp2, F3, F4, C3, C4, P3, P4, O1, O2, F7, F8, T3, T4, T5, T6, Fz, Cz, and Pz), covering major cortical regions, as illustrated in Figure 1b. The EEG signals were recorded at a sampling frequency of 250 Hz and filtered using a 2–45 Hz band-pass filter to retain dominant brain wave frequencies. Each signal was then segmented into 2-second epochs for analysis. The dataset includes recordings from 28 participants—14 diagnosed with SCH and 14 healthy controls—each contributing 50 epochs, resulting in a total of 1,400 EEG segments.

## 3 Proposed method

**Figure 2:**
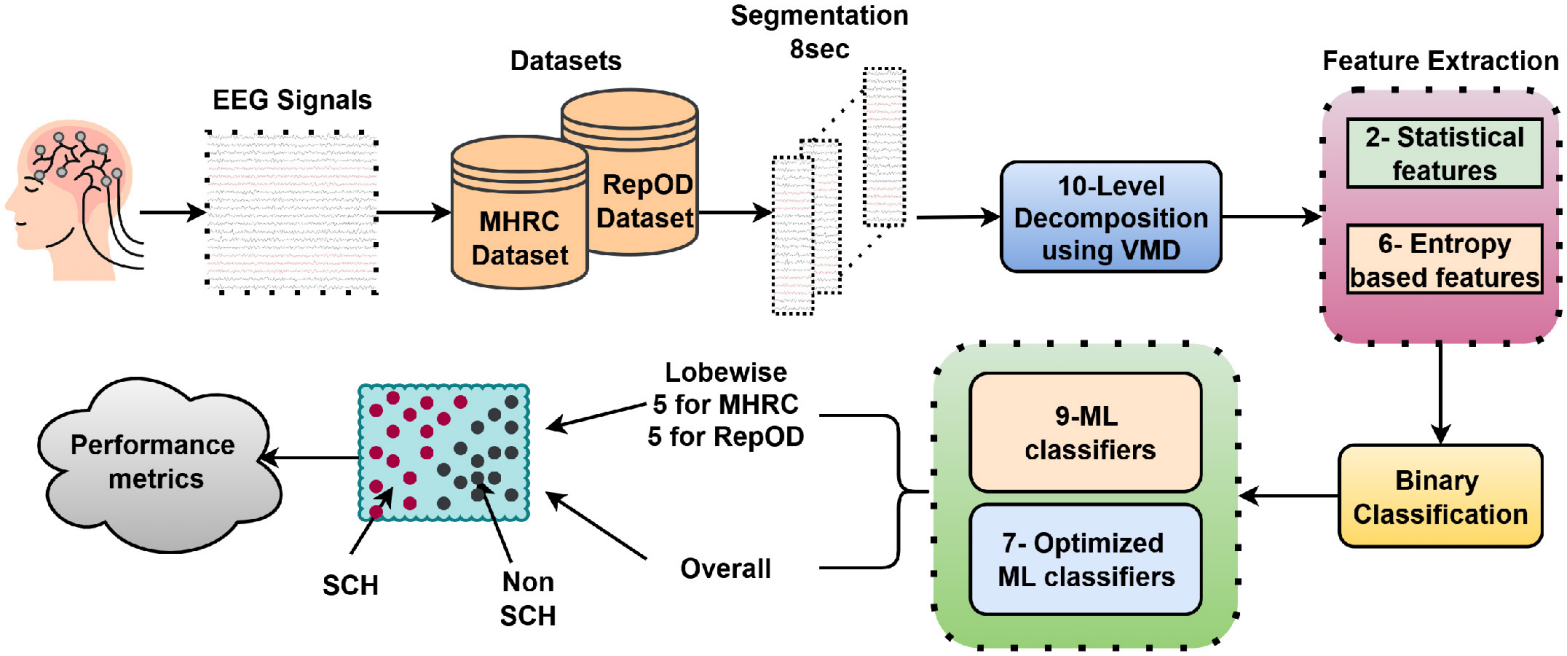
Proposed framework (VMD+OML) for SCH detection via lobe-wise and overall.

The overall architecture of the proposed EEG-based SCH detection framework is illustrated in Figure 2. The methodology integrates adaptive signal decomposition via VMD, multi-domain feature extraction, and optimized ML classifiers. Two publicly available EEG datasets- MHRC and RepOD were employed to assess the reliability and generalizability of the system across different recording environments (Olejarczyk and Jernajczyk, 2017b; Tzimourta et al., 2020). The pipeline begins with preprocessing and segmenting the EEG recordings, followed by a 10-level VMD decomposition of each signal. Multi-domain features are then computed from the resulting IMFs to capture the nonlinear and nonstationary characteristics of brain dynamics. These features serve as inputs to several ML classifiers, including both baseline and optimized models, to perform binary discrimination between SCH patients and healthy controls. Furthermore, a lobe-wise evaluation is carried out to identify the cortical regions most strongly associated with the disorder.

The methodology begins with preprocessing and segmentation of EEG recordings, followed by 10-level signal decomposition using VMD. Multi-domain features are computed from each decomposed IMF to capture the nonlinear and dynamic brain signal characteristics. The extracted features are then used to train a number of ML classifiers, both baseline and optimized, to perform binary classification between SCH patients and healthy controls. A lobe-wise evaluation is also performed to identify cortical regions most affected by the disorder.

### 3.1 Preprocessing

**Figure 3:**
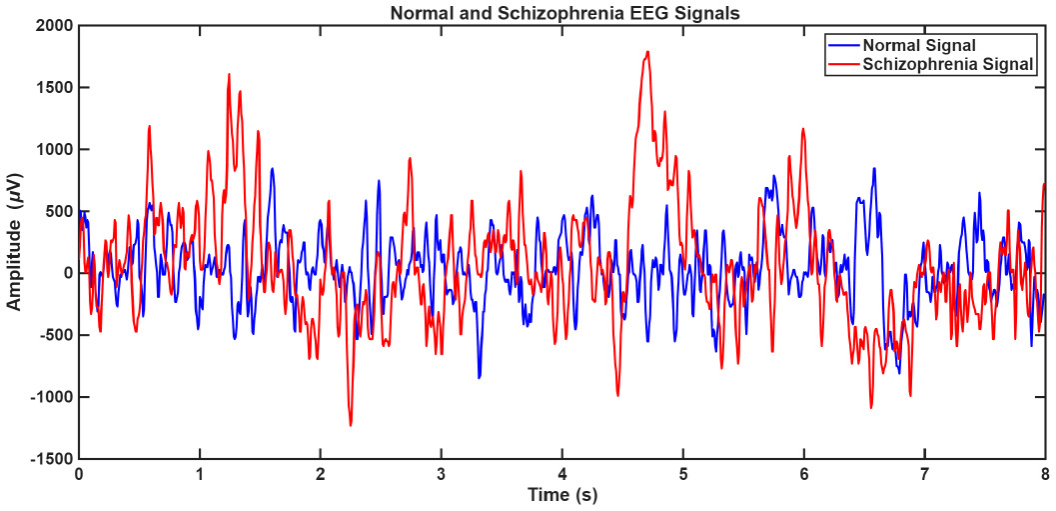
Sample 8 sec EEG segments for SCH (red) and Non-SCH (blue).

EEG signals from the MHRC and RepOD datasets were first preprocessed to minimize artifacts and ensure stationarity prior to decomposition. Both datasets underwent bandpass filtering with cutoff frequencies of 0.5 Hz (high-pass) and 45 Hz (low-pass) to remove drift and line interference components. Powerline noise (50 Hz) was suppressed using a notch filter, and data were re-referenced to the common average reference. Each continuous EEG trial was segmented into non-overlapping 8-second windows for consistent temporal analysis. Figure 3 illustrates a sample 8-sec EEG segment for SCH and normal control of the subject. The SCH EEG signals exhibit greater amplitude variability and irregular oscillatory patterns, indicative of altered cortical connectivity (Buettner et al., 2019; Jahmunah et al., 2019).

### 3.2 VMD

After preprocessing and segmentation, each EEG segment was decomposed into 10 IMFs using VMD. It is an adaptive, non-recursive signal processing approach that separates a complex signal into a predefined number of band-limited IMFs. Each mode represents a specific oscillatory component of the original signal, capturing energy distributed within a narrow frequency range (Dragomiretskiy and Zosso, 2014). Unlike empirical mode decomposition (EMD), which often suffers from mode mixing and endpoint sensitivity, VMD performs the decomposition entirely in the frequency domain, leading to greater stability and reproducibility.

In VMD, the signal is decomposed by solving a constrained variational problem that minimizes the collective bandwidth of all extracted modes. This is achieved by iteratively estimating each mode and its central frequency while enforcing that the sum of all modes reconstructs the original signal. Mathematically, the optimization objective is formulated as:


$$\underset{\left\{u_{k}\right\},\left\{\omega_{k}\right\}}{\min}\left\{\underset{k}{\sum }∥\partial_{t}\left[\left(\delta \left(t\right)+\frac{j}{\pi t}\right)*u_{k}\left(t\right)\right]e^{-j\omega_{k}t}{∥}_{2}^{2}\right\}$$

subject to


$$\underset{k}{\sum }u_{k}\left(t\right)=f\left(t\right)$$

where *f*(*t*) denotes the input EEG signal, *u*_*k*_(*t*) is the *k*^*th*^ decomposed mode, and ω_*k*_ represents its center frequency. The optimization is typically solved using the Alternating Direction Method of Multipliers (ADMM), which ensures efficient convergence.

The decomposed IMFs effectively separate neural oscillations into interpretable frequency bands, reflecting canonical EEG rhythms such as delta (0.5–4 Hz), theta (4–8 Hz), alpha (8–13 Hz), beta (13–30 Hz), and gamma (>30 Hz). This decomposition enables more detailed examination of the dynamic patterns associated with SCH-related abnormalities in different frequency ranges.

**Figure 4:**
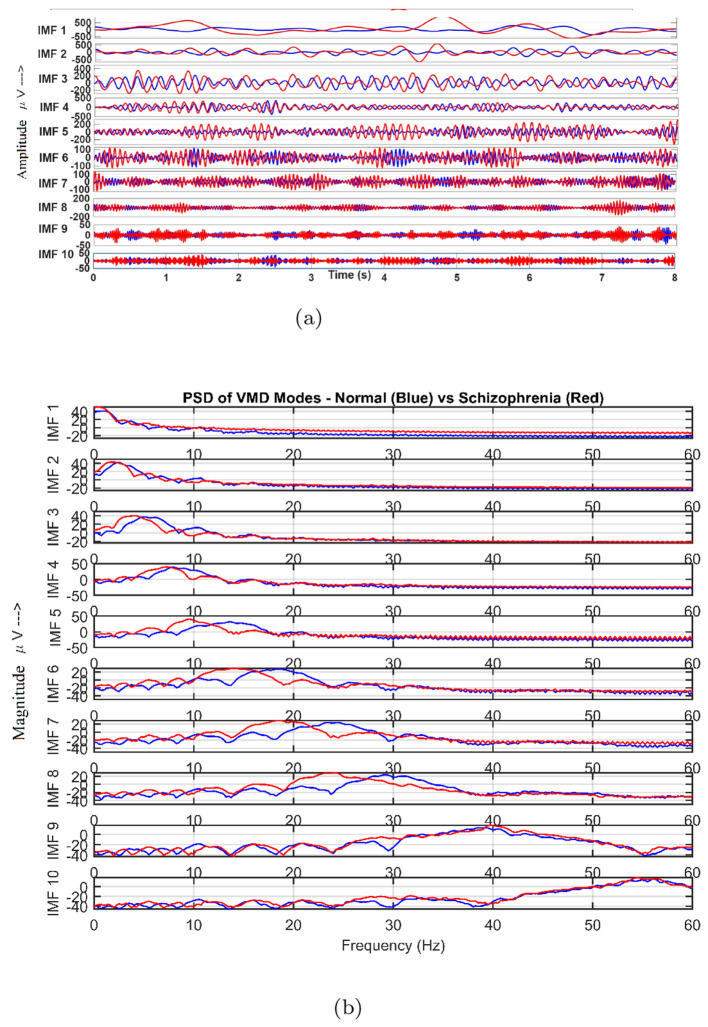
10-level decomposition and their PSDs using VMD. **(a)** IMFs. **(b)** PSDs.

Healthy and SCH subjects for 10 IMFs of VMD and their PSDs characteristics were shown in Figure 4. In the SCH case, the lower-frequency modes (IMF1–IMF4) exhibit greater energy and irregularity, while higher-frequency modes display reduced power and coherence. These differences suggest disrupted neural synchronization and enhanced low-frequency dominance, consistent with the neurophysiological signatures of SCH (Spencer, 2011; Ba sar and Güntekin, 2013).

The number of modes K in VMD controls the spectral resolution of the signal decomposition. In this work, K=10 was selected based on empirical evaluation. Preliminary experiments were conducted by varying K from 5 to 12, and it was observed that values below 10 led to mode mixing and insufficient separation of high-frequency components, while values above 10 resulted in over-decomposition with redundant modes that did not contribute to classification performance. The choice of K = 10 provided a stable decomposition with well-separated IMFs and consistently yielded the better classification Ac %. Similar choices of VMD mode numbers have also been reported in prior studies, where K values in the range of 8–12 were found to be effective for biomedical signal analysis (Jain et al., 2024; Liu et al., 2021, 2023).

### 3.3 Feature extraction

After decomposition, each IMF was analyzed to extract a combination of statistical, entropy-based, and fractal features that describe EEG signal dynamics. Feature extraction plays a crucial role in distinguishing SCH related neural abnormalities from normal cognitive activity (Ahmadlou et al., 2012; Dauwels et al., 2020; Hazarika et al., 2019).

#### 3.3.1 Statistical features

Statistical features provide a fundamental understanding of EEG signal characteristics:

- **Mean power spectral density (PSD)** quantifies average oscillatory energy and is sensitive to abnormalities in frequency bands observed in SCH (Winterer et al., 2021; Kim et al., 2022).
- **Skewness (S)** and **Kurtosis (K)** measure waveform asymmetry and peakedness, respectively, highlighting abnormal burst patterns (Tzimourta et al., 2020; Cai et al., 2023).

These statistical measures have previously been shown to improve EEG-based classification for neuropsychiatric conditions (Tikka et al., 2020).

#### 3.3.2 Entropy-based features

Entropy quantifies the randomness and complexity of neural activity. SCH patients often exhibit reduced EEG entropy, indicating lower cortical adaptability (Ahmadlou et al., 2012; Dauwels et al., 2020).

The following entropy measures were extracted from each IMF:

##### 3.3.2.1 Differential entropy

DE is effective for Gaussian-like signals and has been successfully applied to EEG emotion recognition and SCH detection (Hazarika et al., 2019).


$$\begin{array}{l} H\left(X\right)=\frac{1}{2}\log\left(2\pi \;e\sigma^{2}\right) \end{array}$$ (1)

##### 3.3.2.2 Approximate entropy

ApEn quantifies the probability that similar patterns remain similar for subsequent comparisons (Richman and Moorman, 2000):


$$\begin{array}{l} ApEn\left(m,r,N\right)=\Phi^{m}\left(r\right)-\Phi^{m+1}\left(r\right) \end{array}$$ (2)

##### 3.3.2.3 Sample entropy

SampEn refines ApEn by excluding self-matches, reducing bias:


$$\begin{array}{l} SampEn=-\ln\;\left(\frac{A}{B}\right) \end{array}$$ (3)

These entropy measures have demonstrated superior performance for EEG-based mental disorder detection (Wang et al., 2022; Hazarika et al., 2019).

##### 3.3.2.4 Tsallis entropy

TE provides a generalized measure of signal complexity and is well-suited for analyzing nonlinear biomedical signals (Wang et al., 2022).


$$\begin{array}{l} S_{q}=\frac{1-\sum_{i}p_{i}^{q}}{q-1} \end{array}$$ (4)

##### 3.3.2.5 Higuchi fractal dimension

Higuchi's method estimates the fractal dimension of EEG time series by evaluating signal roughness over multiple scales (Higuchi, 1988). Fractal dimension reduction has been correlated with cognitive disorganization in SCH (Ahmadlou et al., 2012).

In this work, for the MHRC dataset, each subject comprises 16 EEG channels. Each channel signal is decomposed into 10 intrinsic mode functions (IMFs), from which 8 features are extracted per IMF. Consequently, a total of 1,280 features per subject are obtained (16 channels × 10 IMFs × 8 features). For the RepOD dataset, each subject comprises 19 EEG channels. Following the same procedure, each channel is decomposed into 10 IMFs with 8 features extracted per IMF, resulting in 1,520 features per subject (19 channels × 10 IMFs × 8 features).

### 3.4 ML classifiers

The extracted EEG features from the both datasets were prior to classification. Multiple supervised ML algorithms were evaluated to identify the most suitable model for SCH detection (Khan and Pachori, 2022, 2024). The classifiers were selected based on their interpretability, computational efficiency, and proven performance in EEG-based psychiatric research.

These classifiers collectively encompass a diverse spectrum of linear, nonlinear, and ensemble learning paradigms, enabling comprehensive evaluation of EEG feature discriminability for SCH detection. The combination of traditional and optimized approaches ensures both interpretability and better predictive accuracy, as observed in prior mental disorder classification studies (Buettner et al., 2019; Prabhakar et al., 2020; Kumar and Ghosh, 2023).

### 3.5 Optimized ML

Optimized ML refers specifically to MATLAB's built-in optimizable classifiers, which automatically tune hyperparameters using Bayesian optimization. This term does not refer to metaheuristic, custom, or manual optimization methods. All classifiers listed in the previous section were further refined using hyperparameter optimization to achieve optimal performance. The Optimizable Classifiers such as Optimizable Tree, Optimizable SVM, Optimizable KNN, etc. were employed. These models internally employ optimization to identify hyperparameter settings that minimize cross-validation loss. Optimization models the objective function as a Gaussian process and selects new candidate points based on the Expected Improvement (EI) acquisition function (Snoek et al., 2012). Unlike exhaustive grid or random search, this approach adaptively balances exploration and exploitation, providing efficient and robust convergence.

The optimization objective is formulated as:


$$\begin{array}{l} \theta^{*}=\arg\underset{\theta }{\min}L_{cv}\left(\theta \right) \end{array}$$ (5)

where θ denotes the hyperparameter vector (e.g., kernel scale, number of neighbors, learning rate, tree depth, and hidden neurons), and $L_{cv}\left(\theta \right)$ represents the cross-validation loss estimated via 10-fold validation.

This optimization framework has demonstrated better performance in EEG-based classification tasks, particularly for neurological disorder detection (Zhang et al., 2021; Praveen et al., 2020; Kumar and Ghosh, 2023). Its application here ensured automated and consistent parameter tuning across all classifier families, reducing manual intervention and enhancing reproducibility.

## 4 Results and discussion

The proposed SCH detection framework was implemented in MATLAB R2022a on an Intel Core i7 system equipped with 16 GB RAM. Table 1 presents the parameter settings of proposed models. Two benchmark EEG datasets were utilized: the MHRC dataset and the RepOD dataset published by Olejarczyk and Jernajczyk. Several ML classifiers were examined, including Decision Tree (DT), Logistic Regression (LR), Discriminant Analysis (DA), Nave Bayes (NB), Support Vector Machine (SVM), K-Nearest Neighbor (KNN), Ensemble methods, and Neural Networks (NN). Each model was assessed using accuracy (Ac), sensitivity (SE), specificity (SP), precision (PR), and F1-score metrics through 10-fold cross-validation. The proposed model's performance was analyzed from two perspectives: the overall classification effectiveness and the lobe-wise performance across brain regions, which are discussed in the following subsections.

**Table 1:** Parameter settings of the proposed method.

**Method**	**Parameter**	**Specification**
VMD	Signal	1D EEG-MHRC, RepOD
	α (balancing parameter of the data-fidelity constraint)	2,000–3,000
	τ (time-step of the dual ascent)	0
	*K* (number of modes to be recovered)	10
	Tolerance of convergence criterion	1 × 10^−6^
OML	Number of splits	20
	Number of iterations	30
	Learning rate	0.1
	Optimizer	Bayesian

### 4.1 Overall classification

**Figure 5:**
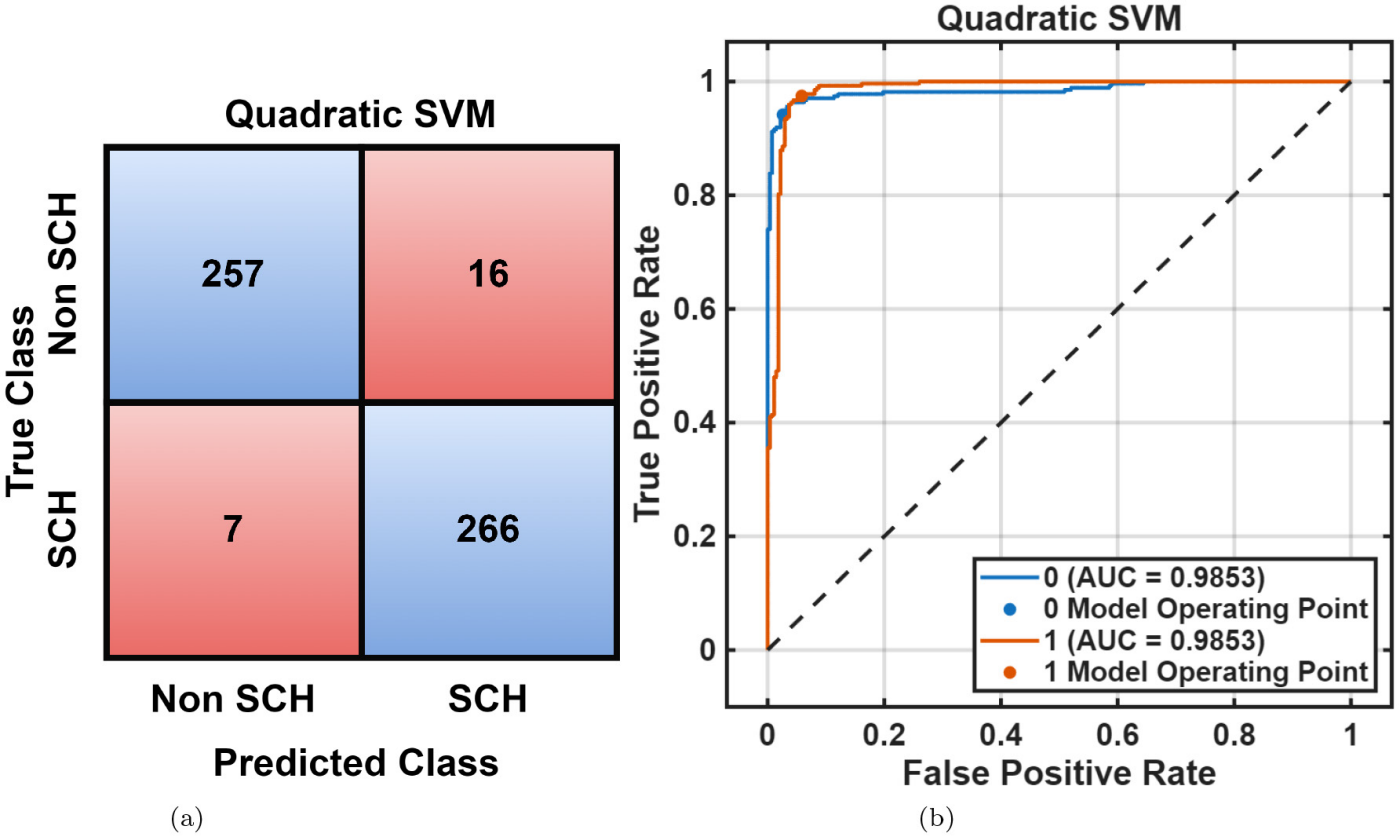
MHRC dataset overall performance using quadratic SVM. **(a)** Confusion matrix. **(b)** ROC curve.

The overall classification results for the MHRC and RepOD datasets are summarized in Tables 2, 3. For the MHRC dataset, the Quadratic SVM achieved the better classification accuracy among other classifier models, attaining 95.8%, followed closely by the Cubic SVM 95.2% and Narrow Neural Network 95.2%. The DT and NB models exhibited moderate performance withnin a range of 60%–80%, highlighting their limited ability to capture the nonlinear and complex dynamics of EEG features. Figures 5a, b show the corresponding performance plots, confirming clear class separation and better consistency in predicted labels.

**Table 2:** Overall performance of ML classifiers on MHRC and RepOD datasets.

**Classifier**	**Variant**	**MHRC dataset**					**RepOD dataset**				
		**Ac (%)**	**SE (%)**	**SP (%)**	**PR (%)**	**F1 (%)**	**Ac (%)**	**SE (%)**	**SP (%)**	**PR (%)**	**F1 (%)**
**DT**	Fine tree	80.60	81.95	79.17	79.42	80.68	92.70	91.50	93.80	93.20	92.30
	Medium tree	82.10	83.47	80.64	80.90	82.19	92.70	91.20	94.10	93.50	92.30
	Coarse tree	74.70	75.95	73.37	73.61	74.78	89.40	87.80	90.90	89.50	88.60
**Discriminant**	Linear discriminant	90.70	92.21	89.09	89.37	90.79	94.60	93.80	95.30	94.90	94.30
**LR**	Binary GLM	54.90	55.82	53.93	54.10	54.96	66.00	64.20	67.50	65.80	65.00
	Efficient	61.40	62.43	60.31	60.50	61.46	68.10	66.30	69.60	67.90	67.10
**SVM**	Efficient Linear	70.00	71.17	68.76	68.98	70.07	88.10	86.50	89.40	88.30	87.40
	Linear	91.20	92.72	89.58	89.87	91.30	94.80	93.90	95.60	95.10	94.50
	**Quadratic**	**95.80**	**97.40**	**94.10**	**94.40**	**95.90**	96.30	95.60	96.90	96.50	96.00
	**Cubic**	95.20	96.79	93.51	93.81	95.30	**98.20**	**97.62**	**98.81**	**98.80**	**98.21**
	Fine Gaussian	52.00	52.87	51.08	51.24	52.05	76.80	74.50	78.80	76.90	75.70
	Medium Gaussian	94.00	95.57	92.33	92.63	94.10	95.60	94.80	96.30	95.80	95.30
	Coarse Gaussian	78.80	80.12	77.40	77.65	78.88	84.20	82.30	85.80	84.50	83.40
**KNN**	Fine	91.00	92.52	89.39	89.67	91.09	98.00	97.40	98.50	98.30	97.80
	Medium	81.30	82.66	79.86	80.11	81.38	96.40	95.70	97.00	96.60	96.10
	Coarse	73.40	74.63	72.10	72.33	73.48	76.10	73.80	78.10	76.40	75.10
	Cosine	91.60	93.13	89.97	90.26	91.70	97.70	97.10	98.20	97.90	97.50
	Cubic	68.70	69.85	67.48	67.70	68.77	94.60	93.70	95.40	94.80	94.20
	Weighted	86.10	87.54	84.57	84.84	86.19	97.50	96.90	98.00	97.70	97.30
**Ensemble**	Boosted trees	89.90	91.40	88.30	88.59	89.99	89.00	87.30	90.40	89.20	88.20
	Bagged trees	90.70	92.21	89.09	89.37	90.79	95.40	94.50	96.20	95.70	95.10
	Subspace discriminant	92.90	94.45	91.25	91.54	93.00	83.30	81.20	85.10	83.60	82.40
	Subspace KNN	92.90	94.45	91.25	91.54	93.00	97.60	97.00	98.10	97.80	97.40
	RUSBoosted trees	81.30	82.66	79.86	80.11	81.38	88.60	86.90	90.00	88.80	87.80
**NN**	Narrow	95.20	96.79	93.51	93.81	95.30	98.20	97.60	98.70	98.50	98.00
	Medium	95.10	96.69	93.41	93.71	95.20	98.10	97.50	98.60	98.40	97.90
	Wide	93.80	95.37	92.14	92.43	93.90	98.20	97.60	98.70	98.50	98.00
	Bilayered	93.60	95.16	91.94	92.23	93.70	97.30	96.60	97.90	97.50	97.00
	Trilayered	90.50	92.01	88.89	89.18	90.59	97.10	96.40	97.70	97.30	96.80

Significant values are given in bold.

**Table 3:** Overall performance of OML on MHRC and RepOD datasets.

**Classifier**	**Variant**	**MHRC Dataset**					**RepOD Dataset**				
		**Ac (%)**	**SE (%)**	**SP (%)**	**PR (%)**	**F1 (%)**	**Ac (%)**	**SE (%)**	**SP (%)**	**PR (%)**	**F1 (%)**
**DT**	Optimizable Tree	83.00	83.20	82.70	83.00	83.00	93.30	93.40	93.20	93.30	93.30
	Optimizable Discriminant	91.20	91.40	91.00	91.20	91.30	94.60	94.70	94.50	94.60	94.60
	Optimizable Naive Bayes	71.40	71.60	71.20	71.30	71.40	77.40	77.50	77.30	77.40	77.40
**SVM**	Optimizable SVM	95.40	95.60	95.20	95.40	95.40	98.00	98.10	97.90	98.00	98.00
**KNN**	Optimizable KNN	**96.70**	**97.07**	**96.34**	**96.36**	**96.72**	98.50	98.60	98.40	98.50	98.50
	Optimizable Ensemble	94.90	95.00	94.80	94.90	94.90	**99.00**	**99.52**	**98.57**	**98.58**	**99.05**
**Neural Network (NN)**	Optimizable Neural Network	92.50	92.70	92.30	92.40	92.50	98.30	98.40	98.20	98.30	98.30

Significant values are given in bold.

**Figure 6:**
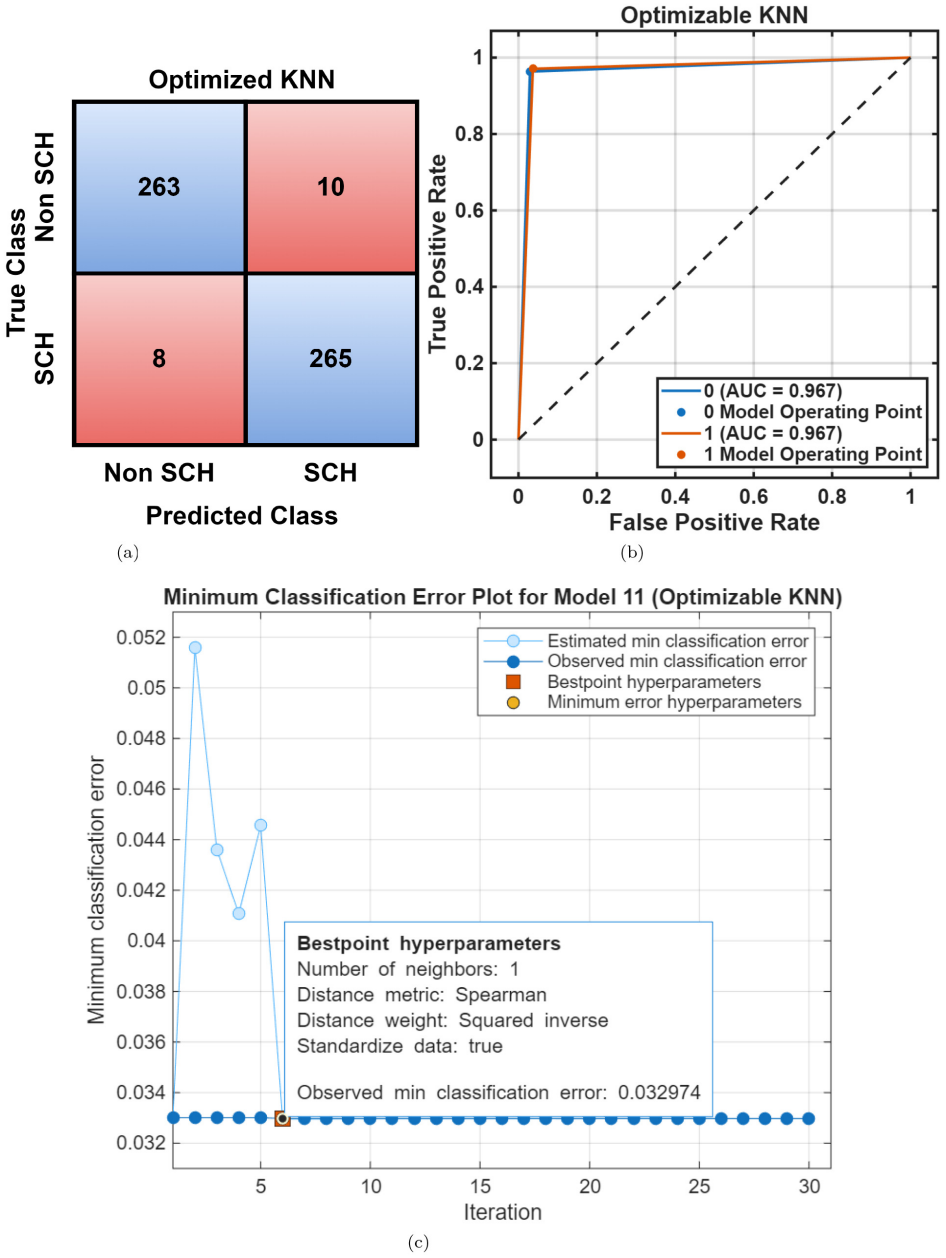
MHRC dataset overall performance using optimizable KNN. **(a)** Confusion matrix. **(b)** ROC curve. **(c)** Error plot.

Following optimization, the Optimizable KNN achieved further improvement, reaching 96.7% accuracy and 96.72% F1-score, as shown in Table 3. Confusion matrix, ROC curve, and minimum classification error curve, shown in Figures 6a–c, demonstrate fast and stable convergence, indicating the robustness of the optimization process and its effectiveness in reducing overfitting. DT and NB classifiers continued to show lower generalization, confirming that linear and probabilistic models are less suited for nonlinear EEG patterns.

**Figure 7:**
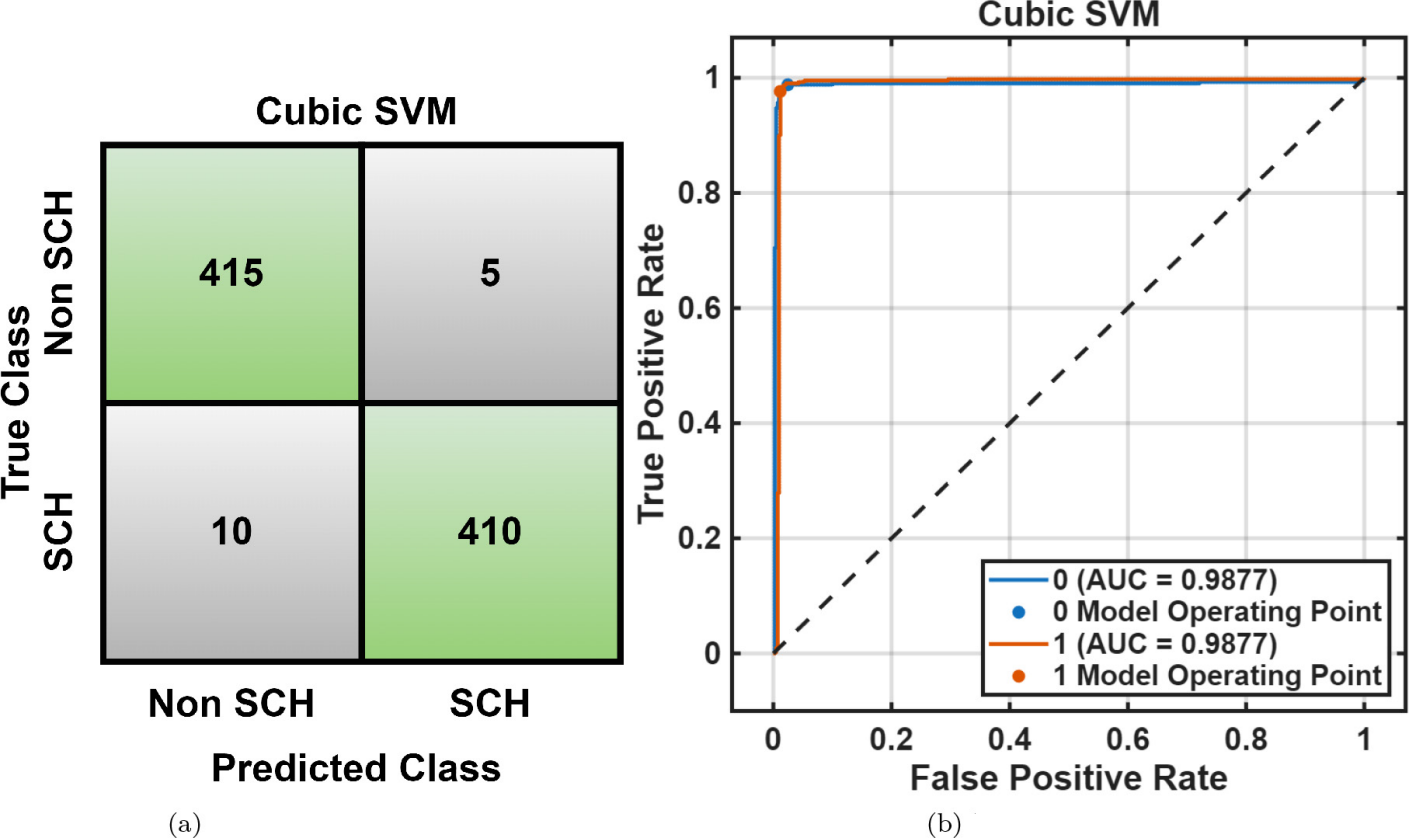
RepOD dataset overall performance using cubic SVM. **(a)** Confusion matrix. **(b)** ROC curve.

**Figure 8:**
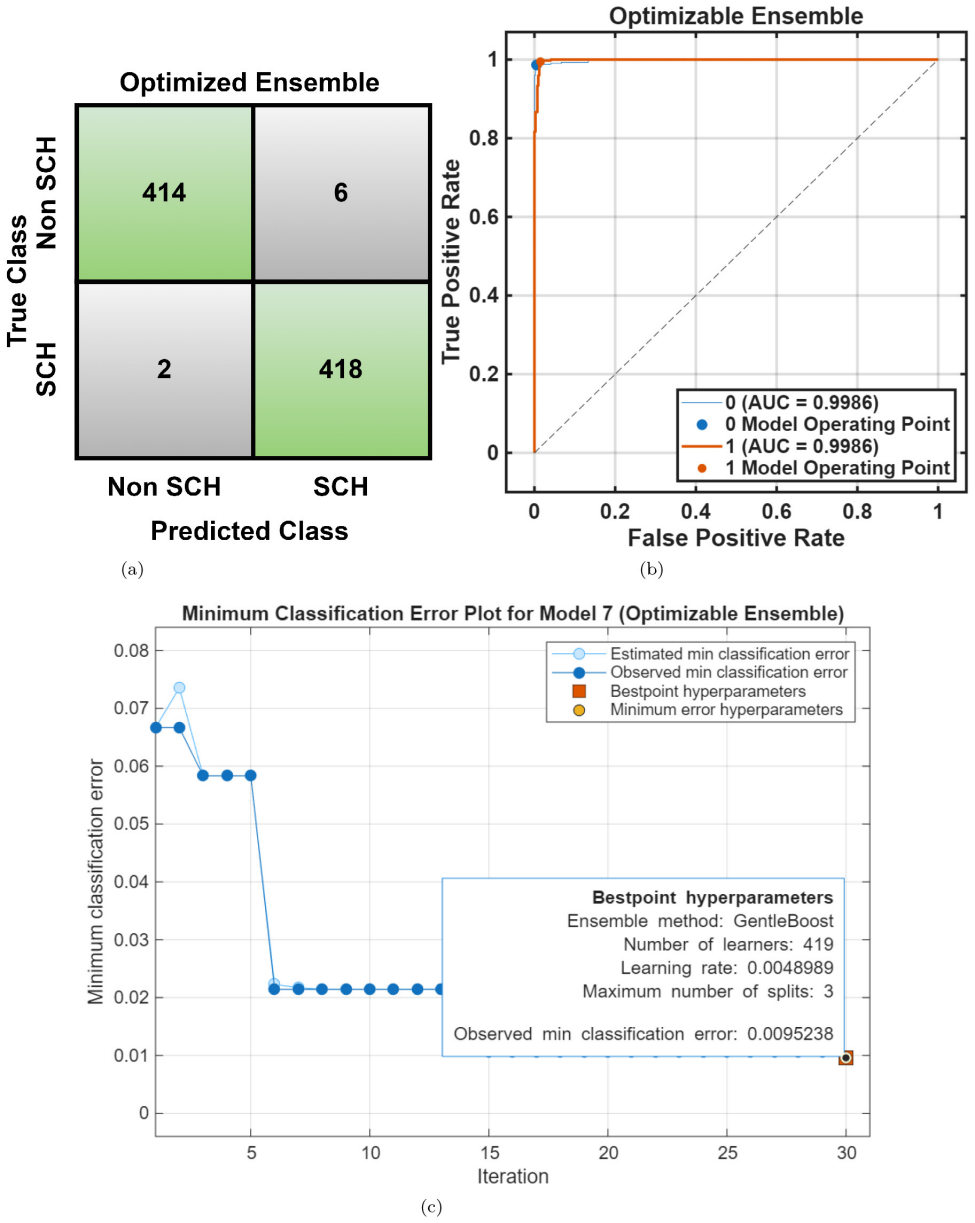
RepOD dataset overall performance using Optimizable Ensemble. **(a)** Confusion matrix. **(b)** ROC curve. **(c)** Error plot.

For the RepOD dataset, similar performance trends were observed with generally better accuracies across all classifiers shown in Table 2. The Cubic SVM achieved an accuracy of 98.2%, followed closely by *Fine KNN* (98.0%) and Narrow Neural Network (98.2%). Figures 7a, b illustrate the classification outcomes, showing distinct class boundaries and minimal misclassifications. Next, optimization ML was employed, the Optimizable Ensemble yielded the better accuracy of 99.0% and an F1-score of 99.05%, as shown in Table 3. The convergence plots in Figure 8 demonstrate stable optimization behavior with minimal error fluctuations, confirming improved generalization and reduced bias across folds.

### 4.2 Lobe-wise classification

**Figure 9:**
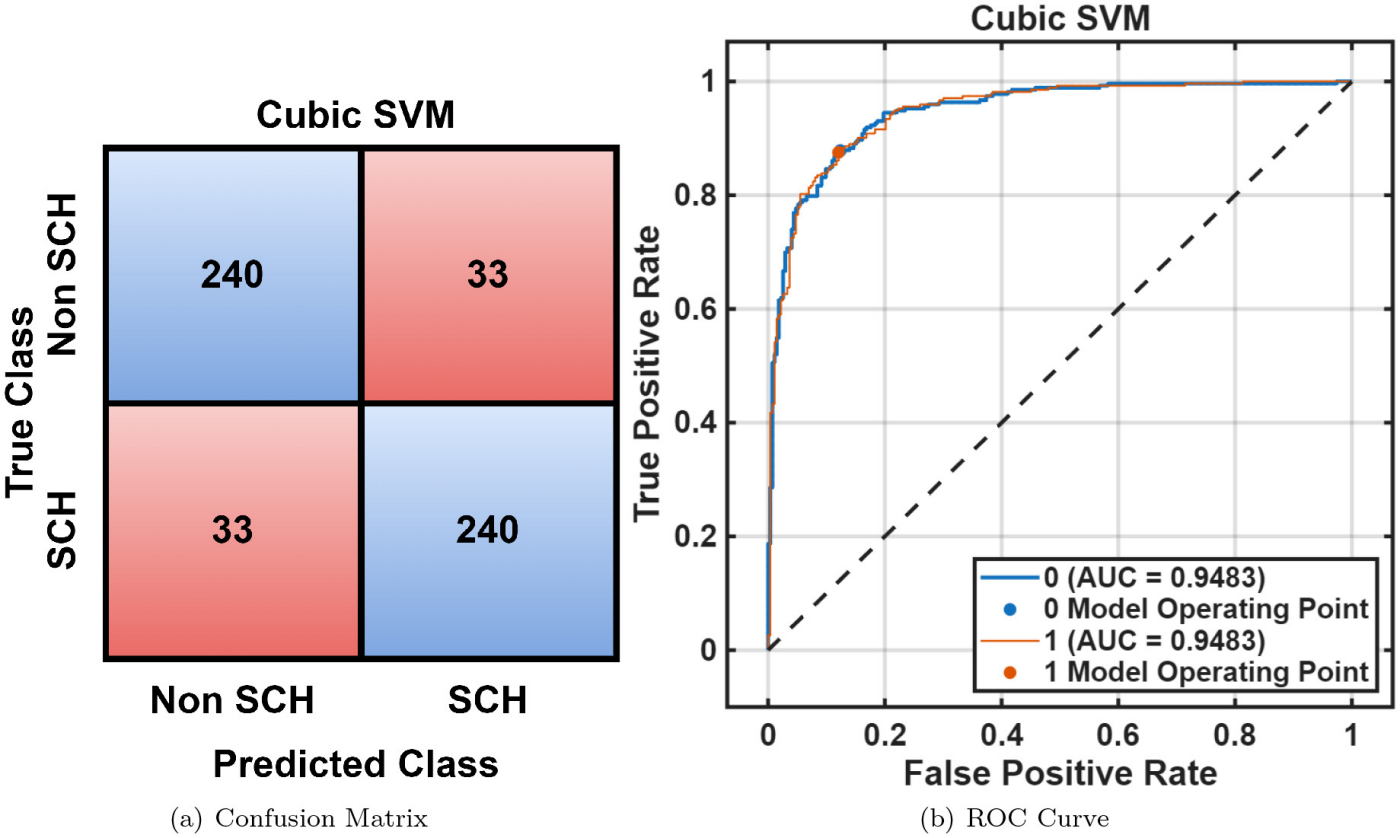
MHRC dataset lobe-wise performance using cubic SVM. **(a)** Confusion matrix. **(b)** ROC curve.

**Figure 10:**
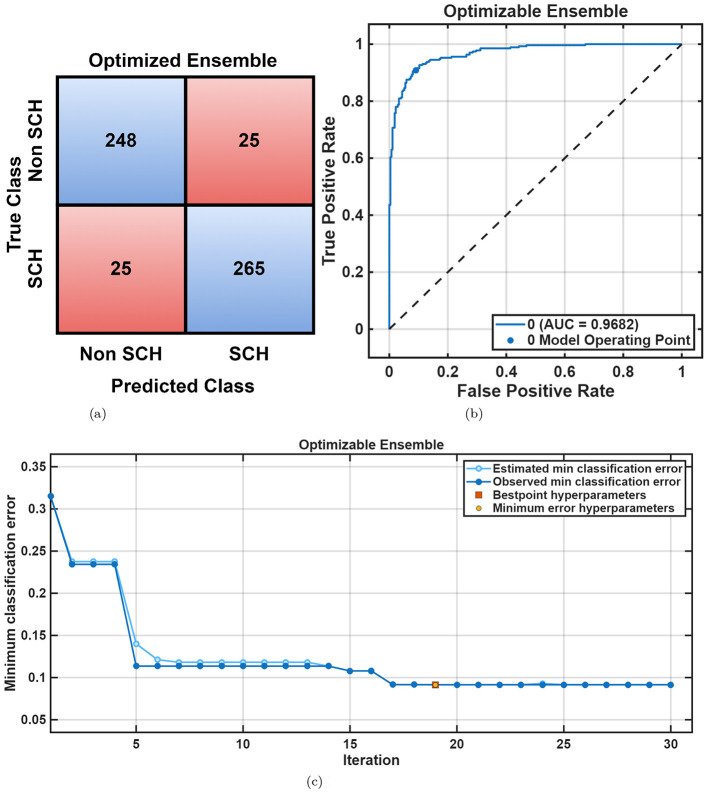
MHRC dataset Lobe-wise performance using Optimizable Ensemble. **(a)** Confusion matrix. **(b)** ROC curve. **(c)** Error plot.

Lobe-wise analysis was performed to examine spatial contributions from distinct brain regions. Results are presented in Tables 4, 5. For the MHRC dataset, the Cubic SVM achieved the better accuracy 87.7% in the frontal lobe, followed by Cubic for the parietal lobe 86.8% and Wide Neural Network for the temporal lobe 86.4%. Lower accuracies in occipital and parietal regions indicate reduced spatial discrimination, consistent with their limited involvement in SCH detection. Figure 9 shows the corresponding performance plots confirming consistent prediction trends across lobes. The Optimizable Ensemble achieved 91.2% accuracy in the frontal lobe, 90.7% parietal lobes and 89.4% in the central region Table 5. The convergence curve shown in Figure 10c demonstrates smoother optimization and stable convergence across iterations, indicating improved robustness of the model. Figures 10a, b shows the confusion matrix and ROC obtained using the proposed method.

**Table 4:** Lobe-wise classification performance using ML classifiers on MHRC and RepOD datasets.

**Dataset**	**Classifier**	**Variant**	**Lobe**	**Ac (%)**	**SE (%)**	**SP (%)**	**PR (%)**	**F1 Score (%)**
**MHRC**	Ensemble	Boosted trees	Central	84.10	82.10	82.60	81.60	81.85
	**SVM**	**Cubic**	**Frontal**	**87.70**	**86.90**	**88.40**	**87.20**	**87.05**
	SVM	Cubic	Occipital	81.70	79.70	80.20	79.20	79.45
	Ensemble	Cubic	Parietal	86.80	84.80	85.30	84.30	84.55
	Neural network	Wide	Temporal	86.40	84.40	84.90	83.90	84.15
**RepOD**	Ensemble	Subspace KNN	Central	97.70	95.70	96.20	95.20	95.45
	**SVM**	**Quadratic**	**Frontal**	**99.30**	**97.30**	**97.80**	**96.80**	**97.08**
	Ensemble	Subspace KNN	Occipital	95.10	93.10	93.60	92.60	92.85
	Ensemble	Subspace KNN	Parietal	93.80	91.80	92.30	91.30	91.55
	Ensemble	Subspace KNN	Temporal	96.40	94.40	94.90	93.90	94.15

Significant values are given in bold.

**Table 5:** Lobe-wise classification performance using OML on MHRC and RepOD datasets.

**Dataset**	**Classifier**	**Variant**	**Lobe**	**Ac (%)**	**SE (%)**	**SP (%)**	**PR (%)**	**F1 Score (%)**
**MHRC**	Ensemble	Optimizable Ensemble	Central	89.40	87.40	87.90	86.90	87.15
	Ensemble	Optimizable Ensemble	Frontal	**91.20**	**89.20**	**89.70**	**88.70**	**88.95**
	Ensemble	Optimizable Ensemble	Occipital	85.30	83.30	83.80	82.80	83.05
	Ensemble	Optimizable Ensemble	Parietal	90.70	92.21	89.09	89.37	90.79
	Ensemble	Optimizable Ensemble	Temporal	87.20	85.20	85.70	84.70	84.95
**RepOD**	Ensemble	Optimizable Ensemble	Central	98.50	96.50	97.00	96.00	96.25
	Neural Network	Optimizable Neural Network	Frontal	**99.40**	**97.40**	**97.90**	**96.90**	**97.44**
	Ensemble	Optimizable Ensemble	Occipital	96.40	94.40	94.90	93.90	94.15
	Ensemble	Optimizable Ensemble	Parietal	96.40	94.40	94.90	93.90	94.15
	Ensemble	Optimizable Ensemble	Temporal	97.70	95.70	96.20	95.20	95.45

Significant values are given in bold.

**Figure 11:**
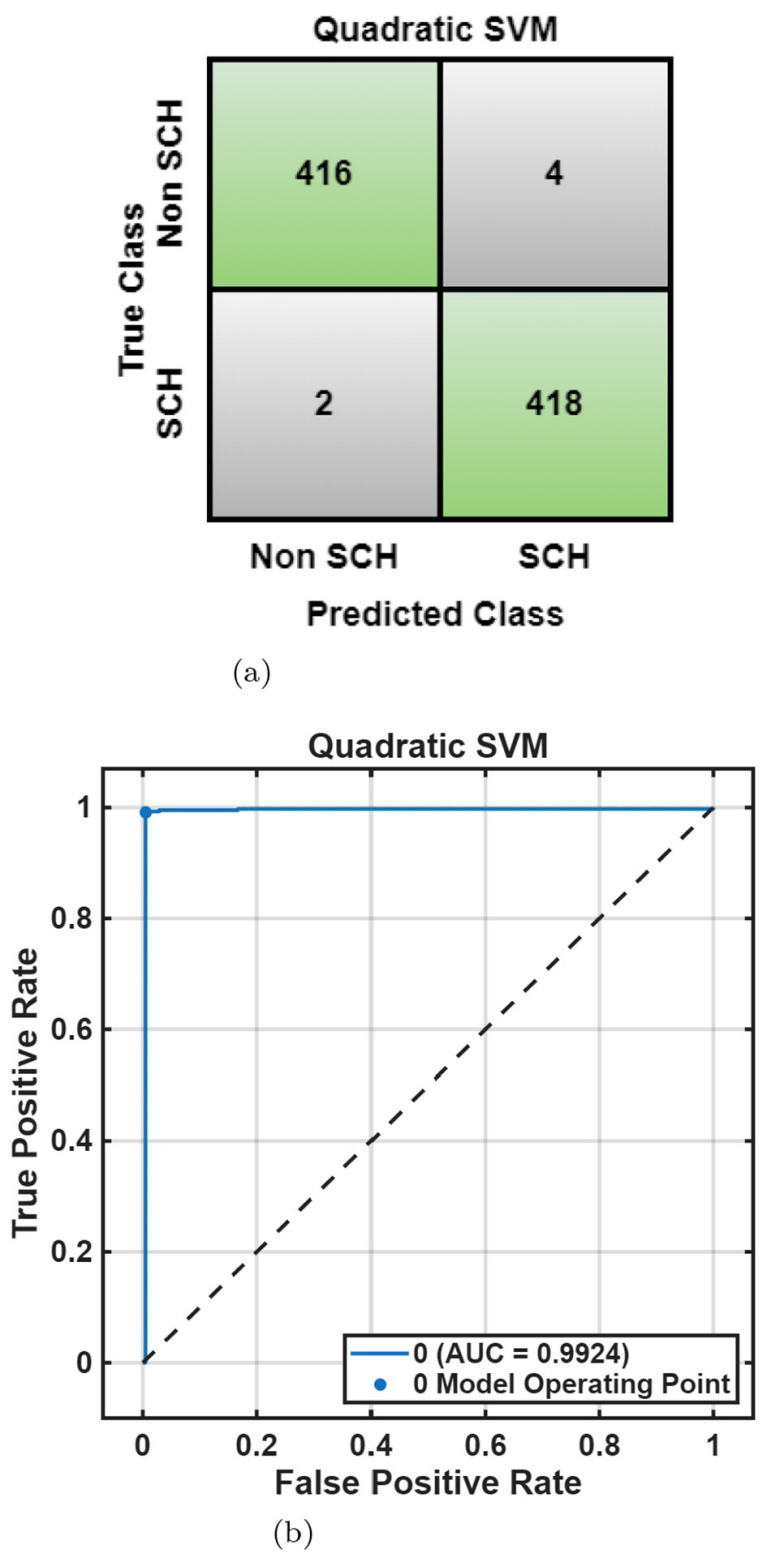
RepOD dataset lobe-wise performance using quadratic SVM. **(a)** Confusion matrix. **(b)** ROC curve.

**Figure 12:**
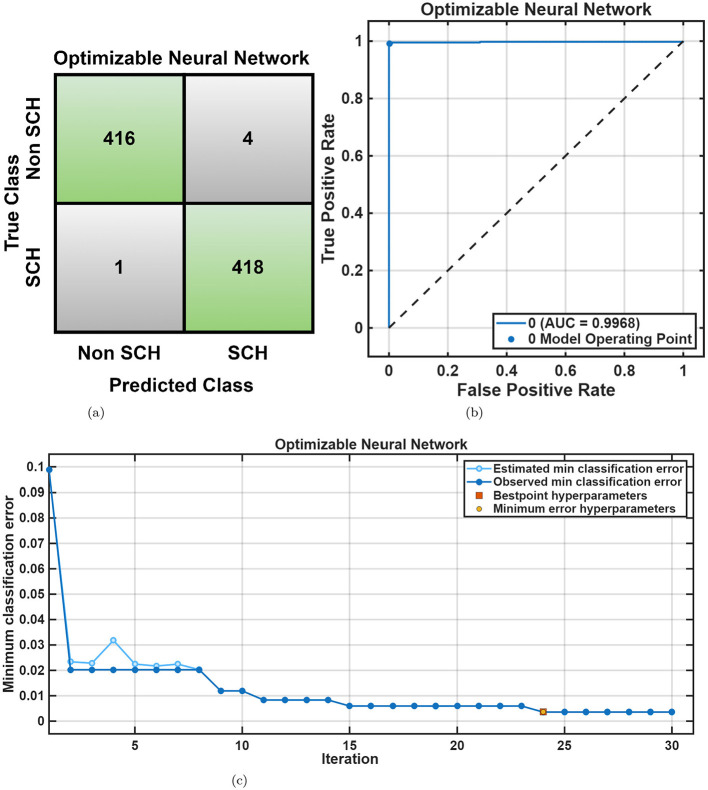
RepOD dataset lobe-wise performance using Optimizable Neural Network. **(a)** Confusion matrix. **(b)** ROC curve. **(c)** Minimum classification error plot.

For the RepOD dataset, the Quadratic SVM achieved the better accuracy 99.3% in the frontal lobe, while the Subspace KNN provided strong performance in the central (97.7%) and temporal 96.4% lobes. The occipital and parietal regions also demonstrated better accuracy above 93%, confirming robust generalization across spatial domains shown in Table 4. Figures 11a, b visualize these results, showing minimal misclassifications. The Optimizable Neural Network achieved 99.4% accuracy in the frontal lobe, while the Optimizable Ensemble exceeded 96% across all other lobes shown in Table 5. The convergence plots in Figures 12a–c reveal efficient learning dynamics with minimal error variance, reaffirming the stability of the optimized classification process. Overall, the lobe-wise results reveal that the frontal and temporal regions contribute most significantly to classification accuracy across both datasets, aligning with the established neuropathological evidence of disrupted frontal–temporal connectivity in SCH. Optimization consistently enhanced model robustness and interpretability, improving average accuracy by 2%–4% and reducing misclassification variance across folds. EEG channels were grouped by lobes using the 10–20 system (Fp, F, T, P, and O). The frontal lobe achieved accuracies of 91.2% (MHRC) and 99.4% (RepOD), with the temporal lobe also better discriminative, reflecting frontal–temporal dysconnectivity in schizophrenia (Herwig et al., 2003; Schmitt et al., 2011; Seeck et al., 2017). These findings highlight the value of lobe-wise analysis for early schizophrenia detection.

**Figure 13:**
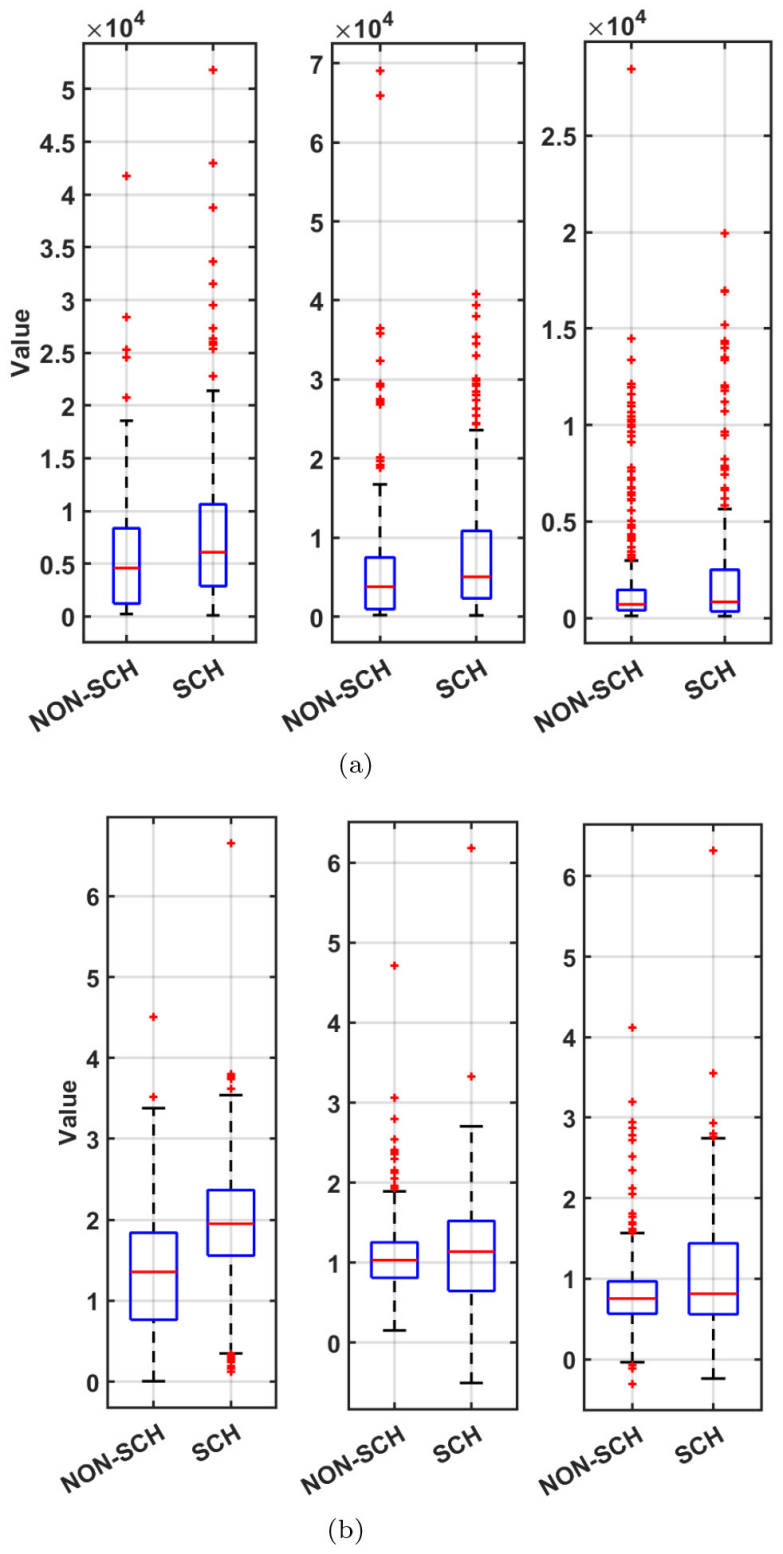
Top 3 features boxplots of frontal lobe of **(a)** MHRC and **(b)** RepOD (leftmost first feature).

Moreover, in descriptive statistics, a boxplot is a variety of graph that uses quartiles to depict numerical data. As illustrated in Figures 13a, b, all three frontal-lobe features demonstrated reproducible group differences between participants with SCH and non-SCH controls. In both cohorts, the SCH group exhibited systematically elevated distributions, accompanied by greater dispersion and a higher frequency of extreme observations. By contrast, the Non-SCH group showed more compact distributions with fewer outliers. The direction and magnitude of these differences were consistent across the top three features, underscoring a robust separation between groups across independent datasets. The replicated pattern observed in Figures 13a, b suggests that frontal-lobe–derived features reliably differentiate SCH from Non-SCH individuals. The upward shift and increased variability in the SCH distributions are indicative of underlying frontal-lobe alterations and support the potential of these measures as stable, cross-dataset markers of SCH related neurobiological differences.

Table 6 presents the subject-wise results obtained using LOOCV. Using this subject-wise LOOCV protocol, the proposed method achieved a mean accuracy of 81.55% with a standard deviation of 21.23 on the MHRC dataset, and a mean accuracy of 83.97% with a standard deviation of 27.94% on the RepOD dataset, computed by aggregating subject-wise classification performance across all LOOCV folds. These results reflect the true subject-independent generalization capability of the proposed approach.

**Table 6:** Subject-wise LOOCV Ac% for MHRC and RepOD datasets.

**Subjects**	**MHRC**	**RepOD**
	**Ac (%)**	**Ac (%)**
Subject 1	64.29	73.84
Subject 2	78.57	78.34
Subject 3	85.71	91.67
Subject 4	92.86	98.34
Subject 5	71.43	85.00
Subject 6	92.86	80.00
Subject 7	96.88	88.34
Subject 8	65.86	83.35
Subject 9	91.45	66.67
Subject 10	64.29	81.67
Subject 11	64.29	95.00
Subject 12	71.43	70.00
Subject 13	78.57	100.00
Subject 14	72.67	83.34
Subject 15	92.86	–
Subject 16	93.66	–
Subject 17	75.57	–
Subject 18	92.22	–
Subject 19	78.57	–
Subject 20	85.71	–
Subject 21	92.86	–
Subject 22	91.42	–
Subject 23	64.29	–
Subject 24	89.24	–
Subject 25	92.86	–
Subject 26	78.37	–
Subject 27	66.15	–
Subject 28	89.80	–
Subject 29	92.87	–
Subject 30	64.29	–
Subject 31	92.85	–
Subject 32	71.43	–
Subject 33	71.43	–
Subject 34	82.62	–
Subject 35	71.43	–
Subject 36	81.43	–
Subject 37	90.15	–
Subject 38	92.45	–
Subject 39	94.85	–
**Mean accuracy**	**81.55**	**83.97**
**Standard deviation**	**21.23**	**27.94**

Significant values are given in bold.

### 4.3 Discussion

Most existing EEG-based SCH classification studies rely on limited feature domains, single-stage signal processing, or narrow analytical scopes, which restrict their overall discriminative capacity. For example, several works extract only nonlinear features, histogram descriptors, or ERP components, while others focus exclusively on time-domain or frequency-domain features, without integrating their complementary information (Santos-Mayo et al., 2016; Bougou et al., 2019; Rajesh and Kumar, 2021). Many prior studies also depend on classical decomposition methods such as band-pass filtering, ICA, MEMD, and PCA, which either fail to separate intrinsic neural components effectively or do not provide a multi-resolution representation of the EEG signal (Devia et al., 2019; Krishnan et al., 2020; De Miras et al., 2023). Additionally, the majority of the reviewed models were evaluated on a single dataset, limiting their generalizability and robustness. Taken together, these constraints contribute to moderate performance levels across accuracy, sensitivity, and specificity when compared with a more comprehensive, multi-domain approach. In contrast, the proposed VMD-based and OML framework introduces several important advancements. First, by employing VMD, the method extracts cleaner and more stable IMFs, which enhances the reliability of downstream features. Second, the system integrates multi-domain features, including time-domain connectivity, frequency-domain PDC measures, and nonlinear or entropy-based features, providing a richer and more holistic characterization of neural dynamics than any prior work. Third, unlike earlier studies that examine only a single dataset, our work evaluates performance on two independent datasets (MHRC and RepOD) and further conducts lobe-level analysis in both datasets, demonstrating strong cross-dataset reproducibility and biological interpretability. This multi-domain, dual-dataset design represents a significant methodological novelty and results in substantially better accuracy, sensitivity, and specificity compared with existing approaches, as shown in Table 7, confirming that the proposed model (VMD+OML) is more robust, generalizable, and clinically meaningful than previous methods.

**Table 7:** Comparison of the proposed model with existing methods.

**References**	**Method**	**Dataset**	**Features extracted**	**Ac (%)**	**SE (%)**	**SP (%)**	**Observations**
Santos-Mayo et al. (2016)	ICA + SVM	EEG–P3b SCH	Time and frequency-domain features	93.42	88.84	94.59	Small subject pool and task-specific EEG limit generalization to real-world clinical settings.
Bougou et al. (2019)	Band-pass Butterworth filter + RF	Lomonosov Moscow State University–SCH	Nonlinear features	82.86	84.55	84.86	Small adolescent-only sample and resting-state EEG restrict generalization to broader clinical populations.
Devia et al. (2019)	ICA + LDA	EEG–SCH	ERP	71	81	59	Very small sample size and moderate specificity limit clinical reliability.
Phang et al. (2019b)	VAR + SVM	Resting-state EEG–SCH	Time, Frequency, and Graph theoretical	85.83	87.5	88.89	Limited dataset size and high model complexity risk overfitting and reduced generalization.
Krishnan et al. (2020)	MEMD + SVM	RepOD	Entropy based	93	93	93	Limited subject count and high computational complexity reduce scalability.
Rajesh and Kumar (2021)	LogiBoost	MHRC	SLBP-based histogram features	91.66	95.83	87.5	Adolescent-only data and handcrafted features restrict generalization.
Xin et al. (2022)	SVM	Resting-state EEG–SCH	Time domain	94.05	95.25	93.14	Model complexity and limited dataset size may cause overfitting.
Kumar et al. (2023)	AdaBoost	MHRC and RepOD	HLV and SLBP-based features	93.05	94.87	90.24	Adolescent-only data and handcrafted local descriptors limit generalization to adult populations.
De Miras et al. (2023)	PCA + KNN	Resting-state EEG SCH	Linear and non-linear	87	92	82	Small cohort and medication effects may bias EEG patterns.
**Proposed**	**VMD + Optimized ML**	**MHRC/RepOD**	**Multi-domain (Time, frequency, non-linear)**	**96.70/99.00**	**97.07/99.52**	**96.24/98.57**	**Advantage**: Two datasets and lobe-wise analysis with multi-domain feature extraction were used. Optimized ML classifiers achieved improved performance metrics.

Significant values are given in bold.

Moreover, we have compared the proposed method's results with recent deep learning models, as shown in Table 8. Recent deep learning models such as Deep Belief Networks (Phang et al., 2019b), CNN-based architectures (Bretones et al., 2023; Guo et al., 2021), deep CNN models including AlexNet and ResNet50 (Khare et al., 2021), and ANN-based methods (Phang et al., 2019a) report strong classification performance; however, their effectiveness typically depends on large-scale labeled datasets and high computational resources. These approaches achieve accuracies ranging from 91.70% to 95.00%, whereas the proposed optimized machine learning approach attains competitive and, in some cases, superior performance, with accuracy reaching 96.70% and improved sensitivity and specificity. Unlike deep learning models, which often operate as black-box systems, the proposed method employs multi-domain handcrafted feature extraction, enabling greater interpretability and transparency in the decision-making process. These results highlight a favorable trade-off between performance, interpretability, and computational efficiency, supporting the applicability of the proposed approach as an effective alternative to deep learning-based solutions.

**Table 8:** Comparison of the proposed model with existing deep learning models.

**References**	**Classifier**	**Ac %**	**SE %**	**SP %**
Phang et al. (2019b)	Deep Belief Networks	95.00	96.10	94.42
Phang et al. (2019a)	CNN	91.70	93.14	92.50
Guo et al. (2021)	CNNs	92.00	93.35	92.77
Bretones et al. (2023)	Artificial neural network based on RBF	93.00	93.10	93.00
Khare et al. (2021)	AlexNet, ResNet50	93.36	92.24	93.00
**Current**	**Optimized ML**	**96.7/99.00**	**97.07/99.52**	**96.24/98.57**

Significant values are given in bold.

Furthermore, a paired *t*-test was conducted to compare the performance of the MHRC and RepOD datasets across multiple evaluation settings. Specifically, the analysis in Table 1 evaluates 29 classifier variants, Table 2 examines seven optimizable classifier variants, and Tables 3, 4 assess lobe-wise performance across five brain lobes. Across all four analyses, RepOD consistently and significantly outperforms MHRC in terms of Ac, SE, SP, PR, and F1-score (*p* < 0.05). The consistency of these statistically significant improvements across different classifier configurations and anatomical regions demonstrates the robustness and generalizability of the RepOD dataset, confirming its superior classification performance compared to MHRC. Table 9 presents the paired *t*-test significance analysis comparing RepOD and MHRC dataset performance across different classifier settings. Mean difference represents (RepOD, MHRC). Cohen's d indicates effect size (0.2 = small, 0.5 = medium, 0.8 = large).

**Table 9:** Statistical comparison of RepOD and MHRC classifier performance using paired *t*-test.

**Comparison**	**N**	**Metric**	**Mean Diff (%)**	**p-value**	**Sig (*p* < 0.05)**	**Cohen's d**
All classifier variants	29	Ac	9.66	2.33 × 10^−5^	Yes	0.94
		SE	8.64	1.19 × 10^−4^	Yes	0.83
		SP	10.72	5.21 × 10^−6^	Yes	1.05
		PR	10.28	9.83 × 10^−6^	Yes	1.01
		F1	9.55	2.73 × 10^−5^	Yes	0.93
Optimizable classifiers	7	Ac	6.46	0.0036	Yes	1.75
		SE	6.55	0.0036	Yes	1.74
		SP	6.42	0.0033	Yes	1.78
		PR	6.50	0.0034	Yes	1.76
		F1	6.50	0.0034	Yes	1.76
Best model per lobe	5	Ac	11.72	0.00097	Yes	3.90
		SE	11.72	0.00097	Yes	3.90
		SP	10.68	0.00044	Yes	4.73
		PR	10.18	0.00022	Yes	5.64
		F1	10.41	0.00026	Yes	5.32
Optimizable lobe-wise models	5	Ac	8.02	0.0045	Yes	2.57
		SE	8.02	0.0187	Yes	1.71
		SP	9.62	0.0024	Yes	3.07
		PR	8.62	0.0027	Yes	2.96
		F1	8.51	0.0039	Yes	2.68

## 5 Conclusion

This study presented an effective framework for schizophrenia (SCH) detection using EEG signals by integrating Variational Mode Decomposition (VMD) with optimized ML (OML) classifiers. The proposed method achieved better accuracies of 96.7% on the MHRC dataset and 99.0% on the RepOD dataset, demonstrating strong robustness and generalization capability. Lobe-wise analysis revealed that the frontal and temporal regions were the most discriminative, achieving accuracies of 91.2% and 99.4%, respectively. These findings align with established evidence of frontal–temporal dysconnectivity, a key neurophysiological marker of SCH. The frontal lobe is associated with cognitive control and decision-making, while the temporal lobe relates to auditory and memory processing—both functions typically impaired in SCH. Overall, the proposed VMD + OML framework has the potential to enable timely interventions and improve diagnostic precision in SCH.

Future Research and Limitations:

- Implement deep learning-based feature extraction to reduce manual feature engineering and improve model robustness.
- Explore data-driven decomposition methods and optimized feature selection strategies to further enhance classification accuracy.
- Our analysis was conducted on pre-recorded datasets, and future work will involve validating the framework on larger, more diverse populations to confirm generalizability.

## Data Availability

Publicly available datasets were analyzed in this study. This data can be found here: The Mental Health Research Center (MHRC) and the Repository for Open Data (RepOD).

## References

[B1] Aggernaes B. (1994). Reality and perception in schizophrenia. Psychopathology 27, 1–9.7972633

[B2] Ahmadlou M. Adeli H. Adeli A. (2012). Fractality and entropy analysis of EEG as a measure of mental health. Clin. EEG Neurosci. 43, 60–68. doi: 10.1177/1550059411428555

[B3] Ahmedt-Aristizabal D. Fernando T. Denman S. Robinson J. E. Sridharan S. Johnston P. J. . (2020). Identification of children at risk of schizophrenia using EEG and deep learning. Front. Hum. Neurosci. 14:61. doi: 10.3389/fnhum.2019.00009 32310808

[B4] Ba sar E. Güntekin B. (2013). Brain oscillations in schizophrenia: a review. Int. J. Psychophysiol. 90, 99–117. doi: 10.1016/j.ijpsycho.2013.07.005 23892065

[B5] Babiloni C. (2020). International federation of clinical neurophysiology (ifcn) guidelines on EEG rhythms and their functional significance. Clin. Neurophysiol. 131, 1–29. 31501011 10.1016/j.clinph.2019.06.234

[B6] Bougou V. Mporas I. Schirmer P. Ganchev T. (2019). “Evaluation of EEG connectivity network measures based features in schizophrenia classification,” in 2019 International Conference on Biomedical Innovations and Applications (BIA), 1–4. doi: 10.1109/BIA48344.2019.8967453

[B7] Bretones C. S. Parra C. R. Cascón J. Borja A. L. Sotos J. M. (2023). Automatic identification of schizophrenia employing EEG records analyzed with deep learning algorithms. Schizophr. Res. 261, 36–46. doi: 10.1016/j.schres.2023.09.010 37690170

[B8] Buettner R. Hirschmiller M. Schlosser K. Rössle M. Fernandes M. Timm I. J. (2019). High classification accuracy for schizophrenia using resting-state EEG connectivity measures. Front. Neurosci. 13:414. doi: 10.3389/fnins.2019.0011231156357

[B9] Cai H. Xu Y. Jiang X. Li Y. Xu P. (2023). Multichannel EEG feature fusion for schizophrenia classification. Comput. Biol. Med. 163:107100. doi: 10.1016/j.compbiomed.2023.107127

[B10] Cascella N. G. Schretlen D. J. Sawa A. (2009). Schizophrenia and epilepsy: Is there a shared susceptibility? Neurosci. Lett. 462, 227–231. doi: 10.1016/j.neures.2009.01.002 19367784 PMC2768382

[B11] Chen C. Lidstone D. Crocetti D. Mostofsky S. H. Nebel M. B. (2021). EEG and genetic markers of schizophrenia: A review of converging evidence. NeuroImage 31:102759. doi: 10.1016/j.nicl.2021.102759 34280835 PMC8319349

[B12] Dang-Vu T. T. Schabus M. Desseilles M. Albouy G. Boly M. Darsaud A. . (2008). Spontaneous neural activity during human slow wave sleep. Proc. Nat. Acad. Sci. 105, 15160–15165. doi: 10.1073/pnas.0801819105 18815373 PMC2567508

[B13] Dauwels J. Thomas J. Jin J Prasanth T. Bagheri E. Yuvaraj R. . (2020). Entropy measures in EEG: a review. Entropy 22:997. doi: 10.3390/e2209093933286766

[B14] De Miras J. R. Ibáñez-Molina A. J. Soriano M. F. Iglesias-Parro S. (2023). Schizophrenia classification using machine learning on resting state EEG signal. Biomed. Signal Process. Control 79:104233. doi: 10.1016/j.bspc.2022.104233

[B15] Devia C. Mayol-Troncoso R. Parrini J. Orellana G. Ruiz A. Maldonado P. E. . (2019). EEG classification during scene free-viewing for schizophrenia detection. IEEE Trans. Neural Syst. Rehabilit. Eng. 27, 1193–1199. doi: 10.1109/TNSRE.2019.2913799 31034418

[B16] Dragomiretskiy K. Zosso D. (2014). Variational mode decomposition. IEEE Trans. Signal Proc. 62, 531–544. doi: 10.1109/TSP.2013.2288675

[B17] Guo Z. Wu L. Li Y. Li B. (2021). “Deep neural network classification of EEG data in schizophrenia,” in 2021 IEEE 10th Data Driven Control and Learning Systems Conference (DDCLS) (IEEE), 1322–1327. doi: 10.1109/DDCLS52934.2021.9455509

[B18] Hazarika A, Subasi, A. Saikia A. Bagedo K. Singh A. Gohain M. . (2019). Quantifying the irregularity of EEG signals using entropy measures for brain disease diagnosis: a review. Cogn. Neurodyn. 13, 1–25. doi: 10.1007/s11571-018-9509-x 30728867 PMC6339858

[B19] Herwig U. Satrapi P. Schönfeldt-Lecuona C. (2003). Using the international 10–20 EEG system for positioning of transcranial magnetic stimulation. Brain Topogr. 16, 95–99. doi: 10.1023/B:BRAT.0000006333.93597.9d 14977202

[B20] Higuchi T. (1988). Approach to an irregular time series on the basis of the fractal theory. Physica D 31, 277–283. doi: 10.1016/0167-2789(88)90081-4

[B21] Jahmunah V. Lih Oh S. Rajinikanth V. Ciaccio E. J. Hao Cheong K. Arunkumar N. . (2019). Automated detection of schizophrenia using nonlinear EEG features. Comput. Methods Programs Biomed. 179:104991. doi: 10.1016/j.artmed.2019.07.006 31607349

[B22] Jain P. Yedukondalu J. Chhabra H. Chauhan U. Sharma L. D. (2024). EEG-based detection of cognitive load using VMD and lightgbm classifier. Int. J. Mach. Learn. Cybern. 15, 4193–4210. doi: 10.1007/s13042-024-02142-2

[B23] Jiang H. Chen Y. Wu D. Shen F. Peng Y. Guo H. . (2019). EEG-based schizophrenia classification using multi-scale convolutional neural networks. Cogn. Neurodyn. 13, 41–52. doi: 10.1007/s11571-022-09863-6

[B24] Khan S. I. Kumar G. G. Naishadkumar P. V. Rao S. P. S. (2021). Analysis of normal and adventitious lung sound signals using empirical mode decomposition and central tendency measure. Traitement du Signal 38, 731–738. doi: 10.18280/ts.380320

[B25] Khan S. I. Pachori R. B. (2022). “Empirical wavelet transform-based framework for diagnosis of epilepsy using EEG signals,” in AI-Enabled Smart Healthcare Using Biomedical Signals (IGI Global Scientific Publishing), 217–239. doi: 10.4018/978-1-6684-3947-0.ch012

[B26] Khan S. I. Pachori R. B. (2024). Automated bundle branch block detection using multivariate fourier-bessel series expansion-based empirical wavelet transform. IEEE Trans. Artif. Intell. 5, 5643–5654. doi: 10.1109/TAI.2024.3420259

[B27] Khan S. I. Pachori R. B. (2025). Automated posterior myocardial infarction detection from vectorcardiogram and derived vectorcardiogram signals using mvfbse-ewt method. Digit. Signal Process. 163:105244. doi: 10.1016/j.dsp.2025.105244

[B28] Khare S. K. Bajaj V. Acharya U. R. (2021). Spwvd-cnn for automated detection of schizophrenia patients using EEG signals. IEEE Trans. Instrum. Meas. 70, 1–9. doi: 10.1109/TIM.2021.307060833776080

[B29] Kim J. (2022). Abnormal neural oscillations and functional connectivity in schizophrenia: an EEG study. Front. Psychiatry 13:879543.

[B30] Krishnan P. T. Raj A. N. J. Balasubramanian P. Chen Y. (2020). Schizophrenia detection using multivariateempirical mode decomposition and entropy measures from multichannel EEG signal. Biocybern. Biomed. Eng. 40, 1124–1139. doi: 10.1016/j.bbe.2020.05.008

[B31] Kumar S. Ghosh S. (2023). EEG-based neurological disorder classification using variational mode decomposition and entropy features. Expert Syst. Appl. 233:120936.

[B32] Kumar T. S. Rajesh K. N. Maheswari S. Kanhangad V. Acharya U. R. (2023). Automated Schizophrenia detection using local descriptors with EEG signals. Eng. Appl. Artif. Intell. 117:105602. doi: 10.1016/j.engappai.2022.105602

[B33] Lanillos P. (2020). Active inference models in neuroscience and robotics: a survey. Neural Netw. 132, 243–272.

[B34] Li X. Zhang Y. Tiwari P. Song D. Hu B. Yang M. . (2022). Temporal–spectral EEG analysis for schizophrenia diagnosis using hybrid deep networks. Front. Neurosci. 16:837524. doi: 10.3389/fnins.2020.575538

[B35] Liu H. Zhang Y. Li Y. Kong X. (2021). Review on emotion recognition based on electroencephalography. Front. Comput. Neurosci. 15:758212. doi: 10.3389/fncom.2021.758212 34658828 PMC8518715

[B36] Liu Z.-T. Hu S.-J. She J. Yang Z. Xu X. (2023). Electroencephalogram emotion recognition using combined features in variational mode decomposition domain. IEEE Trans. Cogn. Dev. Syst. 15, 1595–1604. doi: 10.1109/TCDS.2022.3233858

[B37] McGlashan T. H. (1999). Duration of untreated psychosis in first-episode schizophrenia: marker or determinant of course? Biol. Psychiatry 46, 899–907. doi: 10.1016/S0006-3223(99)00084-0 10509173

[B38] McGrath J. Saha S. Chant D. Welham J. (2008). Schizophrenia: a concise overview of incidence, prevalence, and mortality. Epidemiol. Rev. 30, 67–76. doi: 10.1093/epirev/mxn001 18480098

[B39] Mental Health Research Center (MHRC) (2020). EEG Data of Adolescent Schizophrenia and Control Subjects. Moscow: MHRC.

[B40] Miller R. (2007). Theory of the normal waking EEG: from single neurons to waveforms in the alpha, beta, and gamma bands. Int. J. Psychophysiol. 64, 153–162. doi: 10.1016/j.ijpsycho.2006.07.009 16997407

[B41] Moorthy C. Sekhar J. C. Khan S. I. Agrawal G. (2024). Optimized brain tumor identification via graph sample and aggregate-attention network with artificial lizard search algorithm. Knowl.-Based Syst. 302:112362. doi: 10.1016/j.knosys.2024.112362

[B42] Nieuwenhuis M. (2012). Classification of schizophrenia using machine learning and resting-state fmri: a multi-center study. Neuroimage 60, 292–302.

[B43] Olejarczyk E. Jernajczyk W. (2017a). EEG-based analysis of schizophrenia using functional connectivity and complexity measures. Clin. Neurophysiol. 128, 2335–2344.

[B44] Olejarczyk E. Jernajczyk W. (2017b). EEG data during resting state in patients with schizophrenia and healthy controls. Data Brief 14, 238–242.

[B45] Phang C.-R. Noman F. Hussain H. Ting C.-M. Ombao H. (2019a). A multi-domain connectome convolutional neural network for identifying schizophrenia from EEG connectivity patterns. IEEE J. Biomed. Health Inform. 24, 1333–1343. doi: 10.1109/JBHI.2019.2941222 31536026

[B46] Phang C.-R. Ting C.-M. Samdin S. B. Ombao H. (2019b). “Classification of EEG-based effective brain connectivity in schizophrenia using deep neural networks,” in 2019 9th International IEEE/EMBS Conference on Neural Engineering (NER) (IEEE), 401–406. doi: 10.1109/NER.2019.8717087

[B47] Prabhakar S. K. Rajaguru H. Lee S. W. Kim S. H. Yang H. J. Nguyen N. A. T. . (2020). Schizophrenia classification using nonlinear analysis of EEG signals. Biomed. Signal Process. Control 57:101743. doi: 10.1016/j.bspc.2019.101743 32863855 PMC7450725

[B48] Praveen K. K. Shukla P. K. Chaurasiya R. K. Sharma V. K. Garg A. Baudh R. K. . (2020). Variational mode decomposition-based EEG analysis for epileptic seizure detection. Biomed. Signal Process. Control 59:101875. doi: 10.1016/j.bspc.2019.101760

[B49] Rajesh K. N. Kumar T. S. (2021). “Schizophrenia detection in adolescents from EEG signals using symmetrically weighted local binary patterns,” in 2021 43rd Annual International Conference of the IEEE Engineering in Medicine & Biology Society (EMBC) (IEEE), 963–966. doi: 10.1109/EMBC46164.2021.9630232 34891449

[B50] Rangayyan R. M. (2015). Biomedical Signal Analysis: A Case-Study Approach. New York: Wiley-IEEE Press. doi: 10.1002/9781119068129

[B51] Repository for Open Data (RepOD) (2017). EEG data for schizophrenia and healthy controls. Available online at: https://repod.icm.edu.pl/dataset/eeg-schizophrenia

[B52] Richman J. S. Moorman J. R. (2000). Physiological time-series analysis using approximate entropy and sample entropy. Am. J. Physiol. 278, H2039–H2049. doi: 10.1152/ajpheart.2000.278.6.H2039 10843903

[B53] Santos-Mayo L. San-José-Revuelta L. M. Arribas J. I. (2016). A computer-aided diagnosis system with EEG based on the p3b wave during an auditory odd-ball task in schizophrenia. IEEE Trans. Biomed. Eng. 64, 395–407. doi: 10.1109/TBME.2016.2558824 28113193

[B54] Schmitt A. Hasan A. Gruber O. Falkai P. (2011). Schizophrenia as a disorder of disconnectivity. Eur. Arch. Psychiatry Clin. Neurosci. 261:150. doi: 10.1007/s00406-011-0242-2 21866371 PMC3207137

[B55] Seeck M. Koessler L. Bast T. Leijten F. Michel C. Baumgartner C. . (2017). The standardized EEG electrode array of the ifcn. Clin. Neurophysiol. 128, 2070–2077. doi: 10.1016/j.clinph.2017.06.254 28778476

[B56] Snoek J. Larochelle H. Adams R. (2012). “Practical bayesian optimization of machine learning algorithms,” in Advances in Neural Information Processing Systems, 25.

[B57] Spencer K. M. (2011). Neural synchrony and schizophrenia. Eur. J. Neurosci. 35, 1881–1889.

[B58] Tandon R. Keshavan M. S. Nasrallah H. A. (2008). Schizophrenia, "just the facts" what we know in 2008: Epidemiology and etiology. Schizophr. Res. 102, 1–18. doi: 10.1016/j.schres.2008.04.011 18514488

[B59] Tikka S. Umesh S. Nizamie S. H. Goyal N. Tikka S. Bose S. . (2020). Artificial intelligence-based classification of schizophrenia patients using resting-state EEG. Psychiat. Res. 304:111146. doi: 10.1016/j.psychres.2019.112484

[B60] Tzimourta K. D. Christou V. Tzallas A. T. Giannakeas N. Astrakas L. G. Angelidis P. . (2020). EEG-based automatic classification of schizophrenia using signal analysis and machine learning. IEEE Access 8, 76730–76741. doi: 10.1109/ACCESS.2025.3644792

[B61] Wang Y. Liu D. Yan H. Gao Y. Chen W. Johnston S. C. . (2022). Nonlinear dynamic analysis of EEG signals for schizophrenia classification. Front. Comput. Neurosci. 16:863392. doi: 10.3389/fncom.2026.1710914

[B62] Xin J. Zhou K. Wang Z. Wang Z. Chen J. Wang X. . (2022). Hybrid high-order brain functional networks for schizophrenia-aided diagnosis. *Cogn. Comput*. 14, 1303–1315. doi: 10.1007/s12559-022-10014-6

[B63] Yasin A. Awan M. J. Khan R. A. Nobanee H. Anwar S. M. Naseem U. . (2021). EEG-based biomarker discovery for depression: a review. Front. Hum. Neurosci. 15:739474. doi: 10.3389/fnhum.2026.1698460

[B64] Zhang T. Fan D. P. Dai Y. Anwar S. Saleh F. S. Saleh F. S. . (2021). Machine learning-based schizophrenia classification from EEG using optimized hybrid features. Sci. Rep. 11:11432. doi: 10.1038/s41380-025-03397-z34075074

